# Differential Protein and Glycan Packaging into Extracellular Vesicles in Response to 3D Gastric Cancer Cellular Organization

**DOI:** 10.1002/advs.202300588

**Published:** 2023-06-20

**Authors:** Álvaro M. Martins, Tânia M. Lopes, Francisca Diniz, José Pires, Hugo Osório, Filipe Pinto, Daniela Freitas, Celso A. Reis

**Affiliations:** ^1^ i3S‐Institute for Research and Innovation in Health University of Porto Rua Alfredo Allen 208 Porto 4200-135 Portugal; ^2^ IPATIMUP‐Institute of Molecular Pathology and Immunology University of Porto Rua Júlio Amaral de Carvalho 45 Porto 4200-135 Portugal; ^3^ Instituto de Ciências Biomédicas Abel Salazar (ICBAS) University of Porto R. Jorge de Viterbo Ferreira Porto 4050-313 Portugal; ^4^ Faculty of Medicine of the University of Porto Alameda Prof. Hernâni Monteiro Porto 4200-319 Portugal

**Keywords:** cancer, extracellular vesicles, glycosylation, lectins, proteome

## Abstract

Alterations of the glycosylation machinery are common events in cancer, leading to the synthesis of aberrant glycan structures by tumor cells. Extracellular vesicles (EVs) play a modulatory role in cancer communication and progression, and interestingly, several tumor‐associated glycans have already been identified in cancer EVs. Nevertheless, the impact of 3D tumor architecture in the selective packaging of cellular glycans into EVs has never been addressed. In this work, the capacity of gastric cancer cell lines with differential glycosylation is evaluated in producing and releasing EVs when cultured under conventional 2D monolayer or in 3D culture conditions. Furthermore, the proteomic content is identified and specific glycans are studied in the EVs produced by these cells, upon differential spatial organization. Here, it is observed that although the proteome of the analyzed EVs is mostly conserved, an EV differential packaging of specific proteins and glycans is found. In addition, protein–protein interaction and pathway analysis reveal individual signatures on the EVs released by 2D‐ and 3D‐cultured cells, suggesting distinct biological functions. These protein signatures also show a correlation with clinical data. Overall, this data highlight the importance of tumor cellular architecture when assessing the cancer‐EV cargo and its biological role.

## Graphical abstract

This study reveals differential packaging of glycans and proteins in extracellular vesicles (EVs) in response to changes in cellular architecture. Signatures identified in EVs from 3D tumor cell cultures allow to cluster subgroups of gastric cancer patients with distinct prognostic information. These findings emphasize the importance of tumor cellular architecture when assessing the EV glycosylation, proteome, and biological role.

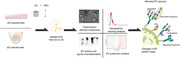

## 1 Introduction

Gastric cancer is the fifth most common and the third most lethal type of cancer, with around one million of new cases and 700 000 deaths occurring in 2020 worldwide.^[^ ^1^ ^]^ Over the years, countless efforts were made to identify novel approaches and models that could improve the diagnostic, prognostic, and survival rate of gastric cancer patients. The use of complex in vitro culture models, such as 3D cancer models, have gained importance in the cancer research field as it showed to provide more accurate biological knowledge of the tumor cells.^[^ ^2^, ^3^, ^4^ ^]^ In particular, the cancer cells grown under 3D cell culture methodologies revealed to better mimic in vivo conditions regarding cellular drug resistance,^[^ ^5^ ^]^ hypoxia,^[^ ^6^ ^]^ substrate distribution,^[^ ^7^ ^]^ and cancer‐associated glycosylation,^[^ ^4^, ^8^ ^]^ when compared to the classic monolayer cell culture.^[^ ^7^, ^9^ ^]^

Importantly, 3D spheroids have already been shown to secrete high levels of functional extracellular vesicles (EVs), whose content and function may differ depending on the culture conditions used.^[^ ^2^ ^]^ EVs are nanoparticles secreted by all cell types that can transport and transfer the cargo (nucleic acids, proteins, lipids, and glycans) of the secreting cell to local and distant sites.^[^ ^9^, ^10^ ^]^ Recent studies have shown that EVs play a key role in dissecting the molecular mechanisms underlying cancer cell communication and can potentially provide biomarkers for diagnosis and cancer patient stratification.^[^ ^11^, ^12^ ^]^ Several studies have shown that, cancer cells use EVs to modulate the behavior of recipient cells and promote tumor progression,^[^ ^12^ ^]^ by inducing pre‐metastatic niches at distant organs.^[^ ^13^ ^]^ Although the role of EVs in cancer biology has been getting a lot of attention, the function of EV‐glycosylation in cancer is far from being fully elucidated. Thus, detailed analysis of EV glycans and their content using adequate cancer cell models in proper culture conditions and EV isolation procedures are key for the transition into functional biological studies.

Protein glycosylation is a post‐translational modification often disturbed in cancer.^[^ ^14^ ^]^ Tumor cells take advantage of these pathways to synthesize aberrant glycans such as the simple truncated *O*‐glycan sialyl‐Tn (STn),^[^ ^15^, ^16^ ^]^ branched *N*‐glycans, and increased sialylated structures that are reported to confer gastric cancer cells with more aggressive phenotypes and associated with gastric cancer patients’ poor survival.^[^ ^17^, ^18^ ^]^ STn is a tumor‐associated antigen expressed in different types of tumors. In particular, it has been reported to be highly expressed in gastric carcinoma,^[^ ^15^ ^]^ where it has been shown to induce a more aggressive phenotype by interfering with key signaling pathways.^[^ ^17^ ^]^ More recently, we have identified this truncated *O*‐glycan in EVs produced by gastric cancer cells.^[^ ^15^, ^19^ ^]^

In this study, we have evaluated the protein and glycan content of EVs secreted from gastric cancer cell lines cultured under conventional 2D and compared with those grown in 3D multicellular tumor spheroid culture conditions. EVs were isolated by the combination of differential ultracentrifugation (UC) and size exclusion chromatography (SEC). Here, we provide evidence that the cell culture architecture affects the packaging of specific cancer‐related proteins and glycans in secreted EVs, with potential biological relevance in clinical data.

## 2 Experimental Section

### 2.1 Cell Culture

The poorly differentiated gastric adenocarcinoma cell line MKN45 WildType (WT) (Japanese Collection of Research Bioresources) displaying a heterogeneous *O*‐glycosylation profile and the previously glycoengineered MKN45 SimpleCell (SC) obtained by targeting the *COSMC* gene by zinc‐finger nucleases to display homogenous truncated *O*‐glycans as previously described^[^ ^20^, ^21^, ^22^ ^]^ were cultured in RPMI 1640 GlutaMAX, HEPES medium supplemented with 10% of fetal bovine serum (FBS) (all from Invitrogen, Carlsbad, CA, USA). Cultured cells were maintained in a cell incubator at 37 °C with 5% CO_2_ and tested for mycoplasma contamination.

### 2.2 3D Multicellular Tumor Spheroid Formation

3D multicellular tumor spheroids formation was performed using 3D Petri Dish (MICROTISSUES technology, MicroTissues Inc., Sigma–Aldrich, St. Louis, MO, USA), in an agarose mold‐based approach, as previously described.^[^ ^4^ ^]^ Micromolds were prepared using 2.4% agarose in 0.9% of NaCl. Before seeding, micromolds were incubated with RPMI for 2 h at 37 °C. A cell suspension of 400 000 cell mL^−1^ was seeded per micromold, and cells were left to settle for 30 min. Finally, additional medium was added to each well. 3D spheroids were maintained in a cell incubator at 37 °C with 5% CO_2_.

### 2.3 Viability Assay

The effect of FBS removal from the cell culture medium was evaluated by trypan blue exclusion assay. For 2D cell culture conditions, cells were plated in 24‐well plates at a density of 5 × 10^4^ cells per well in triplicate. After 24 h, WT and SC cells were washed with PBS and cultured with medium supplemented with 10% or 0% FBS. After 72 h, both cell lines were collected and total cells were counted in a Neubauer chamber using trypan blue (Gibco, Waltham, MA, USA) to distinguish live from dead cells. For 3D cell culture conditions, spheroids were grown in RPMI with 5% FBS for 2 days. The spheroids were washed, and the medium was replaced with either RPMI containing 5% FBS or RPMI containing 0% FBS for a period of 5 days. Finally, spheroids were collected and counted similarly to 2D cells. Two independent experiments with three technical replicates per condition were performed. Results are shown as average ± SD. Unpaired *t*‐test was used for statistical analysis.

### 2.4 Immunofluorescence

The impact of FBS removal on the cellular synthesis of the simple *O*‐glycan STn was tested in all conditions by immunofluorescence. For 2D cell culture conditions, cells were grown in Ibidi slides (Planegg, Germany) and further fixed with 4% w/v paraformaldehyde (Alfa Aesar, Haverhill, MA, USA) at room temperature (RT) for 15 min. For 3D cell culture conditions, spheroids were fixed with 4% w/v paraformaldehyde at RT for 15 min. After fixation, spheroids were covered with Richard–Allan Scientific™ HistoGel™ Specimen Processing Gel (Thermo‐Fisher Scientific) and paraffin‐embedded. Paraffin blocks were cut into 5 µm sections. After deparaffinization and rehydrated, cells were permeabilized with 0.02% Triton^TM^ X‐100 (Sigma–Aldrich, Saint Louis, MO, USA) in PBS for 5 min. Both 2D cells and spheroids were incubated in normal goat serum diluted 1:5 in 10% of BSA for 1 h at RT followed by overnight incubation with the anti‐STn antibody B72.3^[^ ^23^ ^]^ diluted 1:5 in 5% BSA at 4 °C. Alexa 33 Fluor 488 (Life Technologies, Carlsbad, CA, USA) diluted 1:500 in 5% BSA was used as secondary anti‐mouse antibody. Phalloidin CruzFluor^TM^ 594 conjugate (Santa Cruz Biotechnology, Dallas, TX, USA) diluted 1:1000 in 5% BSA for 30 min was used for actin filaments staining and DAPI (Sigma–Aldrich, Saint Louis, MO, USA) for nuclear staining. Images were acquired using a Zeiss Axio Imager Z1 microscope (Zeiss, Oberkochen, Germany).

### 2.5 Extracellular Vesicles Isolation

For 2D cell culture conditions, WT and SC cells were seeded in 150 mm dishes and grown until reaching 80–90% confluency in medium supplemented with 10% FBS. Then, cells were washed three times with PBS for FBS removal and left to grow in RPMI without FBS supplementation for 72 h prior to any EV isolation protocol. Spheroids were cultured in medium with 5% FBS for 2 days, followed by three PBS washes to remove FBS, and then replaced with RPMI without FBS supplementation for 5 days before EV isolation. EVs were isolated by the combination of UC and SEC as previously described.^[^ ^19^ ^]^ Briefly, conditioned media was centrifuged at 800 g for 5 min and at 2 000 g for 10 min, followed by a 0.22 µm pore filtration. Then, media was centrifuged for 20 h (Optima XE 100, SW32 Ti rotor, Beckman Coulter) using thin wall ultra‐clear centrifuge tubes at 100 000 g and 4 °C. A washing step with 0.9% of NaCl was performed for 3 h at 100 000 g and 4 °C. Both 2D‐ and 3D‐derived EV pellets were resuspended in 0.9% of NaCl solution. EVs were then subjected to an extra purification step by SEC using qEV original 70 nm column (Izon, Christchurch, New Zealand), according to manufacturer's instructions. Briefly, the pellet obtained after UC was loaded on top of the qEV column. A total of 15 fractions of 500 µL were collected. EV fractions (7–12) were pulled together to be concentrated by ultrafiltration with 10 kDa Amicon Ultra‐15 centrifugal filter units (EMD Millipore), as it was previously reported.^[^ ^19^ ^]^ EV samples were characterized by nanoparticle tracking analysis (NTA), transmission electron microscopy (TEM), western and lectin blotting, and mass spectrometry.

### 2.6 Nanoparticle Tracking Analysis

NTA was performed to evaluate the size and concentration of EVs according to the manufacturer's instructions using a NanoSight NS300 system (Malvern Technologies, Malvern, UK) configured with a Blue 488 laser to illuminate the particles. Samples were injected with a syringe pump with constant flow injection and three videos of 60 s were captured with 749 frames and camera level at 14–16. The videos were recorded and analyzed with NTA software version 2.3 to determine the size distribution and concentration of the particles through the Stokes–Einstein equation. At least three biological replicates were performed. Results are shown as average ± SD. Unpaired *t*‐test was used for statistical analysis.

### 2.7 Negative Staining Transmission Electron Microscopy

Transmission electron microscopy was performed as previously described.^[^ ^19^ ^]^ Briefly, 5 µL of fresh EV sample solution was mounted on formvar/carbon film‐coated mesh nickel grids (Electron Microscopy Sciences, Hatfield, PA, USA) where EVs were adsorbed for 10 min. The excess liquid was removed with filter paper, and 5 µL of 1% uranyl acetate was added onto the grids for 5 s. This process was repeated three times. EV visualization was carried out on a JEOL JEM 1400 TEM at 120 kV (Tokyo, Japan). The images were digitally recorded using a CCD digital camera (Orious 1100 W Tokyo, Japan).

### 2.8 Western and Lectin Blotting

EV samples were lysed in RIPA lysis buffer supplemented with 1 mm of sodium orthovanadate (Na_3_VO_4_), 1 mm of phenylmethylsulfonyl fluoride (PMSF), and complete protease inhibitor cocktail (Roche, Basel, Switzerland). Western and lectin blotting were performed as previously described.^[^ ^19^ ^]^ Equal amounts of protein were separated by 12% SDS‐PAGE for western blotting and 10% SDS‐PAGE for lectin blotting, followed by transference to nitrocellulose membranes (GE Healthcare, UK). After blocking, membranes were incubated overnight at 4 °C with the following antibodies: anti‐Alix (Cell signaling Technology; clone 3A9, 1:200 dilution), anti‐syntenin‐1 (1:200 dilution, sc‐100336, Santa Cruz Biotechnology Dallas, Texas, EUA), anti‐cytochrome C (1:200 dilution, sc‐100336, Santa Cruz Biotechnology Dallas, Texas, EUA), anti‐HSP70 (1:1000 dilution, EXOAB‐HSP70A‐1; Systems Biosciences, Palo Alto, CA, USA) and anti‐CD9 (1:1000 dilution, EXOABCD9A‐1; Systems Biosciences, Palo Alto, CA, USA). After washing, membranes were incubated for 45 min at RT with the respective secondary antibodies: anti‐mouse (1:5000 dilution, 115‐035‐003, Jackson ImmunoResearch West Grove, Pennsylvania, USA) in 5% milk for syntenin‐1 and cytochrome C or in 5% BSA for Alix; anti‐rabbit (1:25 000 dilution, 111‐035‐003, Jackson ImmunoResearch West Grove, Pennsylvania, USA) in 5% BSA for HSP70 and CD9 antibodies. As a loading control and to assess the total protein profile, a silver staining protocol was performed in parallel using the Pierce™ Silver Stain for Mass Spectrometry (Catalog no.:24 600, Waltham, Massachusetts, USA) as per manufacturer instructions.

For glycan detection, membranes were incubated with an anti‐STn antibody (B72.3 clone described previously,^[^ ^23^ ^]^ 1:3 dilution) and the following biotinylated lectins *Aleuria aurantia* lectin (AAL), *Phaseolus vulgaris leucoagglutinin* (L‐PHA), *Phaseolus vulgaris erythroagglutinin* (E‐PHA), *Vicia villosa* lectin (VVL), and *Sambucus Nigra* lectin (SNA) (all purchased from Vector Laboratories, Burlingame, CA, USA). All lectins were used at 1:2000 dilution, except for L‐PHA, where a 1:1000 dilution was used. An anti‐mouse IgG1 specific was used for STn detection (Jackson ImmunoResearch; 115‐035‐205; 1:25 000 dilution). The Vectastain Elite ABC HRP Kit (Vector Laboratories) was used for lectin recognition, as per manufacturer instructions. The protein bands were visualized by chemiluminescence (ECL) detection reagent (GE Healthcare Life Sciences) and analyzed with the ImageLab software (Bio‐Rad, Hercules, CA, USA). Two independent experiments were performed for the analysis of the protein and glycan profile.

### 2.9 Sample Preparation for Mass Spectrometry Analysis

Each sample was processed for proteomic analysis following the solid‐phase‐enhanced sample‐preparation (SP3) protocol and enzymatically digested with Trypsin/LysC as previously described.^[^ ^24^ ^]^

### 2.10 Proteomic Data Analysis

Protein identification and quantitation was performed by nanoLC‐MS/MS following an already published procedure.^[^ ^25^ ^]^ Proteomic data was analyzed using Proteome Discoverer 2.5.0.400 software (Thermo Scientific). For protein identification, the UniProt database was considered for the *Homo sapiens* (2021_03 with 20371 entries) and *Bos taurus* reviewed Proteomes (2021_03 with 6014 entries) together with a human spectral library (NIST_Human_Orbitrap_HCD_20 160 923). The resulting protein list was filtered to exclude all bovine and other protein contaminants, as well as to only include proteins with at least two unique peptides identified. The identified proteins were checked for the presence of EV markers by matching with the top 1000 identified proteins from Vesiclepedia database (release 15/08/2018), in which some of these markers were highlighted. Cancer‐related proteins were chosen by matching with The Cancer Genome Atlas – Stomach Adenocarcinoma (TCGA‐STAD) data and highlighting some of the proteins known to be relevant in the cancer setting. For quantitative analysis, the 3D/2D protein ratio of all proteins detected was calculated following a pairwise ratio‐based approach. To assess differentially expressed proteins, a *t*‐test (background based) hypothesis test was performed for all identified proteins. Only proteins were considered with the ‐Log_10_(FDR adjusted *p*‐value) above 1.3, and a Log_2_(3D/2D ratio fold change) below −1 for enriched in 2D conditions or above 1 for enriched in 3D conditions, and all values calculated were plotted in a Volcano plot. Additionally, the proteins identified by the software were only considered for quantitative analysis if present in all three replicates. Protein‐protein interaction (PPI) analysis for each dataset was performed using the search tool for retrieval of interacting genes (STRING) tool (version 11.5). To construct the PPI networks, Cytoscape (version 3.9.1) was used, and active interaction sources included experiments, databases, and co‐expression, with a minimum required interaction score of 0.7 (high confidence). Considering the small scale of both 2D and 3D differentially enriched datasets, no more than 10 interactors were added to each network. For clustering analysis, Cytocluster, a plugin for Cytoscape, was used with the following parameters: minimum size = 4, minimum density = 0.05, and edge weights = combined_score. Finally, proteins in each network were verified if it was an enriched protein (shape with outline), a protein present in the EV (shape without outline) or a protein not present altogether (shape with faded color).

### 2.11 In Silico Gastric Cancer Patient Clustering

The gene RNA‐seq expression data of the corresponding proteins found to be upregulated in 2D‐ and 3D‐derived EVs were extracted from the The Cancer Genome Atlas (TCGA) data base (STAD, PanCancer Atlas dataset).^[^ ^26^, ^27^ ^]^ The data are presented as Log‐transformed mRNA expression z‐scores compared to the expression distribution of all samples (log RNA Seq V2 RSEM). Considering that MKN45 has been previously reported to showcase molecular features characterized of the chromosomal instability (CIN),^[^ ^28^ ^]^ only patients with this molecular subtype were evaluated, for a total of 223 samples. To construct the heatmap, the Next‐Generation Clustered Heat Map builder was used.^[^ ^29^ ^]^ Both genes (rows) and patients (columns) were hierarchically clustered based on Euclidean distance metric and Ward agglomeration method. Cumulative survival probabilities of each patient cluster were calculated using the Kaplan–Meier method (univariate log‐rank test) using GraphPad prism v9.4.1.

### 2.12 Quantitative Real‐Time Polymerase Chain Reaction of Glycosyltransferases

Total RNA extracts from cell lysates were obtained using TRIZOL Reagent (Life Technologies). RNA (3 µg) was converted into cDNA using the SuperScript IV Reverse Transcriptase (Invitrogen) according to the manufacturer's instructions. *FUT2*, *FUT3*, *FUT4*, and *FUT8* mRNA expressions were quantified through quantitative real‐time polymerase chain reaction (qRT‐PCR) using SYBrGreen Master Mix (NZYTech). Target mRNA levels were normalized to *β*‐actin housekeeping gene. Primer sequences are listed in Table S1 (Supporting Information). The acquired data have been calculated using the ΔCT approach.

## 3 Results

### 3.1 Characterization of 3D Cell Culture Methodology Suitable for EV Isolation

**Figure 1:**
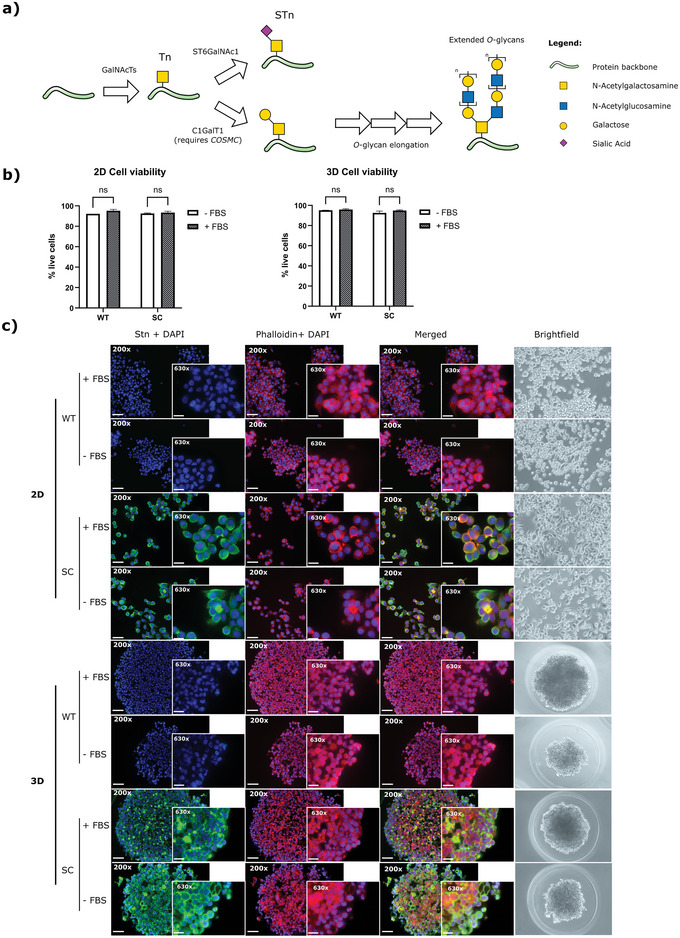
Impact of FBS removal on cell viability and morphology, and STn synthesis in MKN45 WT and SC cell lines cultured under 2D and 3D conditions. a) Schematic representation of STn synthesis. Abrogation of COSMC, a C1GalT1 dedicated chaperone, results in accumulation of truncated *O*‐glycans, Tn, and STn. b) Comparison of cell viability of WT and SC cells when cultured under 2D (left panel) or 3D (right panel) conditions in the presence (+FBS) or absence (‐FBS) of FBS. Results are shown as average ± SD. Student *t*‐test was used for statistical analysis (ns *p*‐value > 0.05). c) Immunofluorescence images of cells grown in the presence (+FBS) or absence (‐FBS) of FBS and stained with anti‐STn antibody B72.3 (green) and phalloidin (red). DAPI (blue) was used for nuclear staining. Images were taken using 200× and 630× amplification, and scale bars represent 50 and 20 µm, respectively. Brightfield images were taken using 200× amplification for 2D‐cultured cells and 100× amplification for 3D‐cultured cells.

In this study, we evaluated and compared the protein and glycan profile of gastric cancer EVs derived from cells cultured under two different cell culture methodologies: the conventional 2D cell culture and 3D multicellular tumor spheroids formation. We used the gastric cancer cell line MKN45 WT displaying a heterogeneous *O*‐glycosylation profile and the genetically engineered MKN45 SC cell line showing homogeneous expression of the truncated *O*‐glycans.^[^ ^20^, ^21^, ^22^ ^]^ Particularly, the SC cell line overexpresses STn (**Figure** **1**a), which have been previously shown to confer gastric cancer cells more aggressive phenotypes.^[^ ^17^, ^30^ ^]^ Considering that FBS contains high levels of glycosylated proteins that can interfere with EV downstream analysis,^[^ ^19^, ^31^ ^]^ we evaluated the effect of FBS removal in cell viability, morphology, and STn synthesis on both gastric cancer cell lines cultured in 2D and 3D conditions (Figure 1). Our results show no significant changes regarding cell viability when both 2D cells and 3D spheroids were grown in the absence of FBS (Figure 1b). In addition, FBS removal did not have an impact on the cellular expression of STn (Figure 1c). As expected, STn expression was only detected in SC cells in both 2D and 3D conditions (Figure 1c). As we have previously reported, cells acquired a more elongated phenotype after FBS removal when cultured in 2D conditions, which was more pronounced in SC cells^[^ ^19^ ^]^ (Figure 1c). In the 3D conditions, the absence of FBS led to the formation of smaller, irregular, and less aggregated spheroids, reflected by their relatively low circularity (Figure 1c). Therefore, as cell viability and STn detection were not affected by FBS removal, EVs were isolated in the absence of FBS from both 2D and 3D cells.

### 3.2 Characterization of EVs Isolated from 2D‐ and 3D‐Cultured Cells

**Figure 2:**
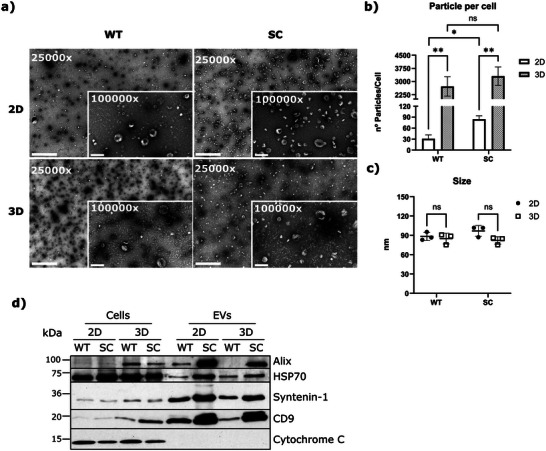
Characterization of EVs isolated from 2D cells and 3D spheroids. a) Representative images of TEM acquired using 25 000× magnification with scale bar representing 1000 nm and zoomed pictures using 100 000× magnification with scale bar representing 200 nm. b) Number of isolated EVs per cell from both WT and SC cells when grown under 2D or 3D conditions. Results are shown as average ± SD. Student *t*‐test was used for statistical analysis (*p‐value < 0.05; ^**^ *p*‐value < 0.01). c) Size mode of isolated EVs from WT and SC cells when grown under 2D or 3D conditions. Results are shown as average ± SD. Student *t*‐test was used for statistical analysis. d) Protein detection of 2D and 3D‐cultured WT and SC cells and corresponding isolated EVs. Western blotting of Alix, HSP70, syntenin‐1 and CD9 as EV markers and cytochrome C as a mitochondrial marker.

Next, we isolated EVs by combining UC with SEC, as we previously showed to be an efficient approach to enrich pure EV samples for proper glycosylation and protein downstream analysis.^[^ ^19^ ^]^ 2D‐ and 3D‐derived EV samples were characterized by NTA and TEM (**Figure** **2**). A significant increased secretion of EVs was measured when cells were grown in 3D culture conditions, in comparison to 2D culture, independently of the glycosylation profile of the cell line (Figure 2b). Additionally, an average of 100 nm of diameter was determined by NTA and a cup shaped EV morphology was visualized by TEM (Figure 2a,c). Additionally, the NTA analysis revealed a higher number of particles per cell in the SC when compared to the WT; however, this was only statistically significant in 2D conditions.

EV markers such as Alix, HSP70, syntenin‐1, and CD9 were detected in our EV samples by western blot (Figure 2d). Additionally, all EV‐samples revealed the absence of the mitochondria marker cytochrome C (Figure 2d). In parallel, we performed a silver staining of all samples to evaluate the protein profile and as a loading control (Figure S1, Supporting Information). Overall, our results show that the combination of UC and SEC methodologies allowed the proper isolation of EV samples from both 2D and 3D cell culture conditions as demonstrated by NTA, TEM, and by the detection of classic EV markers in all the tested conditions.

### 3.3 Cellular Tridimensional Organization Impacts the Glycosylation Profile of the Derived EVs

**Figure 3:**
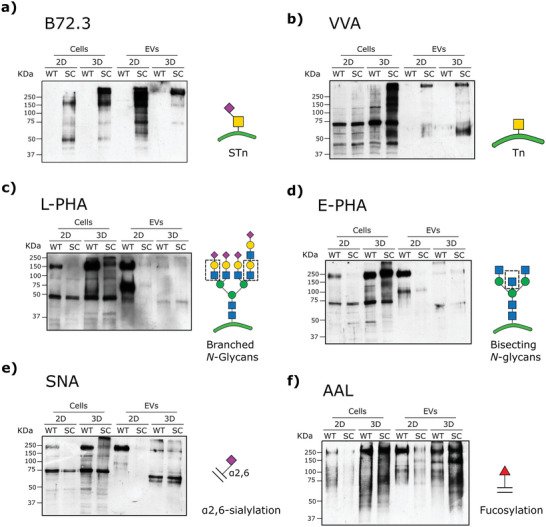
Glycan profile of 2D and 3D‐cultured WT and SC cells and derived EVs. Western blotting using a) an anti‐STn antibody (B72.3) and b) VVL, c) L‐PHA, d) E‐PHA, e) SNA, and f) AAL lectins. Abbreviations: sialyl‐Tn (STn), *Phaseolus vulgaris erythroagglutinin* (E‐PHA), *Phaseolus vulgaris* leucoagglutinin (L‐PHA), *Sambucus Nigra* lectin (SNA), *Vicia villosa* lectin (VVL), and *Aleuria aurantia* lectin (AAL).

The glycan profile of gastric cells cultured under 2D or 3D conditions and their respective isolated EVs were analyzed using the antibody B72.3 for *O*‐glycan STn detection and a panel of lectins for the detection of *O*‐glycan Tn and *N*‐glycan structures (Figure **3**). Interestingly, the different culture conditions affected the general protein band profile of both cells and EVs. The simple *O*‐glycan STn was detected in SC cells and their respective EVs, independently of the cellular architecture. No STn was detected in WT cells and derived EVs (Figure 3a). A more intense band signal between 50 and 250 kDa was detected in SC 3D spheroids, compared to 2D SC cells. Our results also showed an enrichment of STn in 2D SC‐derived EVs when compared to 3D SC‐derived EVs (Figure 3a). We then used the VVL to evaluate the expression of the Tn antigen (Figure 3b), which serves as a precursor for STn formation. Although our results showed a high intensity band signal of Tn in SC spheroids, the predominant form found in SC‐derived EVs was the sialylated structure STn (Figure 3b).

Using a panel of lectins to detect branched and bisecting *N*‐glycans (detected by L‐PHA and E‐PHA, respectively), *α*2,6‐sialic acids (detected by SNA) and fucosylated glycans (detected by AAL), we compared the glycosylation status of cells and EVs harvested from 2D and 3D culture conditions (Figure 3c,d). Our results showed that both WT and SC cells cultured under 3D conditions exhibited higher reactivity to the complex *N*‐glycans (L‐PHA and E‐PHA), as well as to *α*2,6‐sialylated (SNA) and fucosylated structures (AAL), when compared to cells grown in 2D conditions (Figure 3e,f). Remarkably, cellular architecture seemed to impact not only the glycans that were produced by cells, but also the differential packaging of glycans into EVs. We observed that 2D‐derived EVs reflected the lectin profile of 2D cultured cells (Figure 3e,f). However, this effect was not observed in 3D, where low expression of complex *N*‐glycans and *α*2,6‐sialylated structures were detected in 3D‐derived EVs compared to their respective parental cells (Figure 3d–f). On the other hand, regarding AAL reactivity, overall fucosylation motifs were detected at a higher extent in both WT and SC 3D‐derived EVs when compared to 2D‐derived EVs (Figure 3g). These observations can be explained due to an increase in *FUT2* and *FUT8* expression in 3D‐cultured cells evaluated through qRT‐PCR assays (Figure S2, Supporting Information). Altogether, our results demonstrated that changes in the cellular architecture have an impact in the glycosylation status of secreted EVs.

### 3.4 Differential Protein Cargo in Extracellular Vesicles from 2D and 3D Cell Culture Conditions

**Figure 4:**
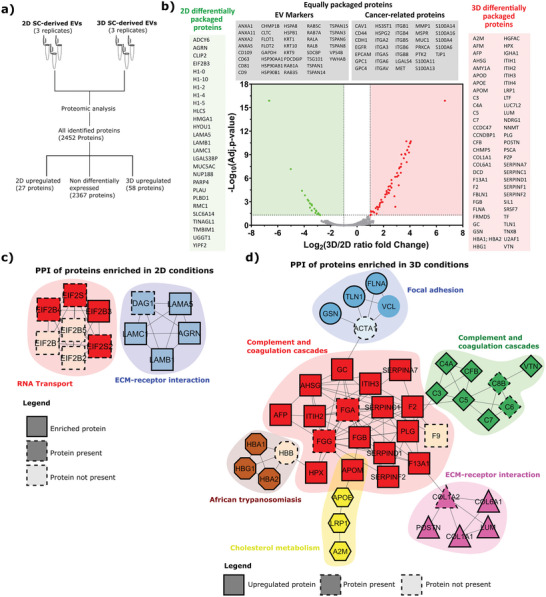
Proteomic and pathway analysis of proteins identified from 2D or 3D SC‐derived EVs. a) Schematic representation of the strategy used for proteomic analysis. Filters were applied to consider a minimum of two unique peptides per identified protein, discard bovine proteins, and exclude common contaminants resulting in 2452 identified proteins. b) Volcano plot of all the 2542 identified proteins. Each protein was distributed according to their Fold Change (3D/2D ratio) and significance level (FDR adjusted *p*‐value) in logarithmic scale. For differentially enriched proteins in 2D conditions (Green dots) and 3D conditions (Red dots) a threshold of Log2(FC) lower than −1 and higher than 1 was used, respectively. Additionally, ‐Log10(FDR adjusted *p*‐value)>1.3 was used as a significance threshold. Proteins packaged at similar levels are represented in grey dots, where some relevant proteins in cancer and EV markers were highlighted in the grey boxes. Only the proteins identified in all replicates and matching the significance and ratio threshold criteria were included for quantitative analysis. c) Analysis of the 2D‐ and d) 3D‐enriched EV proteins for protein‐protein interaction (PPI) network using STRING tools with corresponding KEGG pathway. For PPI analysis, each color represents a cluster of proteins that are functionally connected in the condition under study. c) The PPI analysis of proteins enriched in 2D conditions revealed the clusters related to RNA transport (red) and extracellular matrix (ECM)‐receptor interaction (blue). d) The PPI analysis of proteins enriched in 3D conditions revealed the clusters related to the complement and coagulation cascades (red and green), focal adhesion (blue), African trypanosomiasis (brown), cholesterol metabolism (yellow), and ECM‐receptor interaction (pink). Shapes with solid outline represent proteins enriched in each conditions. Shapes with dashed outline represent proteins detected but not enriched in respective EV samples. Shapes with dashed outline and faded color represent proteins that were not detected in EVs from c) 2D conditions or d) 3D conditions but described to belong to the cluster identified.

Considering the potential impact of STn in EV protein cargo^[^ ^32^ ^]^ and its high relevance in the clinical context of gastric cancer,^[^ ^17^, ^18^, ^33^, ^34^ ^]^ we selected EVs isolated from SC cells cultured under 2D and 3D conditions to quantitatively evaluate its profile using mass spectrometry‐based proteomics approach (Figure **4**a). A total of 2452 proteins were identified, of which 2367 proteins were detected similarly in both samples (Figure 4a). In addition, 27 proteins were detected as differentially enriched in 2D SC‐derived EVs (Figure 4b, green box) and 58 proteins differentially enriched in 3D SC‐derived EVs (Figure 4b, red box). The proteins identified as non‐differentially expressed were marked in the volcano plot as gray dots (Figure 4b). Matching our total list of proteins against the top 1000 proteins identified from Vesiclepedia database (database release 15/10/2018), we detected several EV markers that were enriched in both samples, including tetraspanins (CD9, CD81, and CD63), SDCBP (syntenin‐1), and PDCD6IP (Alix) (Figure 4b). The analysis also revealed numerous cancer‐related proteins annotated in the TCGA‐STAD (the cancer genome atlas stomach – adenocarcinoma) dataset (Figure 4b). Next, we performed protein‐protein interaction (PPI) analysis of enriched proteins in each condition (Figure 4c,d). Considering that EVs are vehicles for cell‐cell communication, we also included in this analysis potential interactor proteins that could be present in recipient cells even if not enriched or present in our datasets EVs. These were highlighted as follows: Enriched proteins as outlined shapes with full color; present but not enriched proteins as dashed outline shapes with full color; and not present proteins as dashed outline shapes with faded color. PPI analysis demonstrated that only a small set of proteins revealed to be capable of interaction with each other in two small clusters. According to the KEGG pathway analysis, these clusters of proteins relate with ECM‐receptor interaction and RNA transport.

Regarding the PPI network of proteins enriched in 3D conditions, a denser set of interactions was observed (Figure 4d). Moreover, most of the proteins enriched in EVs from 3D conditions form functionally connected clusters that participate in processes such as complement and coagulation cascades, focal adhesion, cholesterol metabolism, and extracellular matrix (ECM)‐receptor interaction. These results showed that, although a common protein signature was identified in the EVs isolated from different culture conditions, a differential enrichment of specific proteins was identified when EVs were isolated from 3D cultures.

### 3.5 Enriched Proteins in 3D‐Derived EVs Revealed a Gene Signature Prevalent in Gastric Cancer Patients with Worse Survival

**Figure 5:**
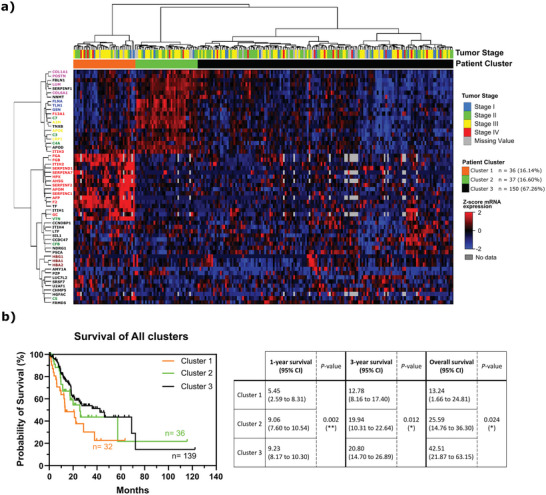
Analysis of the transcriptomic profile from gastric cancer patients according to the proteins enriched in 3D‐derived EVs. a) Heatmap of the transcriptomic profile of gastric cancer patients according to the proteins enriched in 3D‐derived EVs. Only gastric cancer patients classified by the TCGA as chromosomal instability (CIN) molecular type were used, due to the CIN molecular characteristics found in MKN45 cell line. The z‐score for mRNA expression and the covariate scale are represented on the right of the heatmap (missing values = gray). b) Kaplan–Meier survival analyzes patient clusters 1, 2, and 3 (data from STAD, TCGA). The survival curves were significantly different according to the univariate log‐rank test for 1 year (^**^ *p*‐value < 0.01), 3 year (*p‐value < 0.05), and overall survival (*p‐value < 0.05).

Then, we analyzed the transcriptomic data from TCGA‐STAD to assess any possible clinical relevance of the differential enriched proteins identified in each condition (Figure **5**). To perform this analysis, we focused only on patients classified as chromosomal instability (CIN) regarding gastric cancer molecular type to construct the heatmap (Figure 5a), as MKN45 presents characteristics that most closely resembles the CIN subtype.^[^ ^28^ ^]^ The genes corresponding to proteins enriched in EVs from 2D conditions did not show evident clusters in the heatmap (Figure S3, Supporting Information). On the other hand, corresponding genes to the proteins identified as enriched in EVs from SC spheroids formed 3 main clusters across the CIN STAD patients (Figure 5a). Therefore, our analysis defined patient cluster 1 that showed overexpression of genes of proteins contained in the protein cluster related to the complement and coagulation cascade of PPI analysis enriched in EVs isolated from spheroids (Figure 5a). This set of proteins included some of the SERPIN family, tissue factor (TF), and ITIH proteins. The patient cluster 2 (Figure 5a) included a group of STAD patients that overexpresses three other protein clusters identified in PPI analysis, namely a variety of collagen proteins (in pink), TLN1 and FLNA (in blue), and some lipoproteins (in yellow) represented in Figure 4d. The third patient cluster represents the individuals with low expression of the above‐mentioned clusters of proteins. Finally, we compared the 1 year, 3 year, and overall survival rate of all patient clusters. The survival curves of each patient cluster are significantly different among each other (*p*‐values of 0.002(^**^); 0.012(*), and 0.024(*) for 1 year, 3 year, and overall survival, respectively). From the identified patient clusters, patient cluster 1 displayed the lowest 1 year, 3 year, and overall survival median (patient cluster 1 survival median in years of 5.45; 12.78; 13.24 for 1 year, 3 year, and overall survival, respectively), highlighting a possible biological implication for this overexpressing gene signature (Figure 5b). Patient cluster 2 and 3 displayed similar 1‐year and 3‐year survival median; however in overall survival, patient cluster 2 presented a lower median than patient cluster 3 (Figure 5b). Here, we show that patients with overexpression of genes from proteins identified enriched in 3D‐derived EVs related to the complement and coagulation cascade have a relatively poor survival. Overall, these findings suggest a relevant gene signature that can potentially provide biological and prognostic information. These data warrant further validation in independent clinical cohorts.

## 4 Discussion

Aberrant glycan structures synthesized by tumor cells play a role in cancer progression, including the promotion of tumor cell invasion and metastasis, angiogenesis, and modulation of the immune response.^[^ ^34^, ^35^, ^36^, ^37^, ^38^ ^]^ Additionally, the clinical and functional impact of aberrant glycosylation in cancer has been assessed previously in numerous reports.^[^ ^17^, ^30^, ^39^, ^40^, ^41^ ^]^ As important cell‐cell communication vehicles, EVs transport functional molecules, including nucleic acids, lipids, proteins, and glycans.^[^ ^10^ ^]^ In the cancer context, tumor derived‐EVs can modulate and reprogram recipient cells and are important players in inducing pre‐metastatic niche formation and in triggering, or suppressing immune responses.^[^ ^13^, ^42^ ^]^ Considering the prominent role of glycans in modulating cell‐cell interactions in cancer, the cancer EV‐glycosylation constitutes a key component for study, either for biomarker discovery or for the understanding of pathological mechanisms. Therefore, unveiling the tumor‐associated glycans being transported in cancer EVs would significantly contribute to better understanding the role of EVs in this disease. Our group has previously reported that different EV isolation methods influence the enrichment of specific EV subpopulations displaying distinct glycosylation profiles from gastric cancer cells.^[^ ^19^ ^]^ Moreover, it has been described that 3D cell culture promotes packaging of miRNA cargo that should better resemble in vivo EVs,^[^ ^43^ ^]^ and even altering its metabolomic signatures.^[^ ^44^ ^]^ However, to the best of our knowledge, the effects of 3D cellular architecture on the glycosylation profile of secreted EVs have never been reported. In this work, we highlight the differences in the proteomic and glycan profile of EVs isolated from gastric cancer cells depending on the cultured condition. For that, WT cells displaying elongated *O*‐glycans, and the glycoengineered SC that homogeneously displays truncated *O*‐glycans,^[^ ^20^ ^]^ were subjected either to 2D or 3D cellular configuration.

The analysis of EVs isolated by UC combined with SEC revealed that 3D cultured cells produced more EVs per cell when compared to their 2D counterparts as observed in previous studies.^[^ ^2^ ^]^ These results constitute evidence that cellular architecture has a role in EV secretion and is an important factor when studying EV function. It is known that increased EV production is often associated with highly metastatic and aggressive cancer cells.^[^ ^45^, ^46^, ^47^ ^]^ As 3D culture methodologies have been reported to better recreate the biology of tumor, EVs derived from 3D cell cultures may also better recapitulate the in vivo phenotypes. Furthermore, our results revealed higher levels of EV markers in SC EVs obtained from both 2D and 3D conditions. This observation is consistent with prior findings by our group in 2D conditions^[^ ^19^ ^]^ and could be related to the increase EV production, we found in SC cells. Moreover, our proteomic data revealed the presence of LGALS3BP, CALR, MAN2A1, HEXB, and GANAB, proteins previously identified as being enriched in exomere subpopulations,^[^ ^48^ ^]^ in EVs derived from SC under both 2D and 3D conditions. Notably, LGALS3BP exhibited a pronounced enrichment in EVs from cells cultured in 2D conditions, together with an identification of smaller particles in our TEM. Given the significance of glycosylation in EV biology,^[^ ^10^, ^49^ ^]^ it is plausible that the presence of truncated *O*‐glycosylation could potentially affect EV biogenesis. Despite the differences in quantitative EV secretion, similar sizes and morphology were observed in EVs isolated from 2D cells and 3D spheroids.

In this work, we used lectins that are proteins with glycan‐binding affinities and a STn glycan‐binding antibody to assess the glycosylation profile of 2D‐ versus 3D‐derived EVs. We observed that SC cells cultured under 3D conditions have an increased STn expression, together with increased levels of the precursor Tn structure, when compared to 2D conditions. The higher degree of detection of STn and Tn in SC spheroids compared to the 2D counterpart highlights the importance of this type of glycans when changing cell architecture. The Western blot analysis uncovers a wide spectrum of proteins spanning from 50 to 250 KDa, providing further evidence of the upregulation of numerous O‐glycosylated proteins in 3D‐cultured cells.^[^ ^4^, ^50^ ^]^ Nevertheless, EVs derived from SC 3D spheroids showed less STn in specific bands when compared with EVs isolated from 2D cells. While 2D‐derived EVs display STn carriers with a wide range of different molecular weights, 3D‐derived EVs mainly exhibit STn in high molecular weight carriers. This suggests different protein carriers of STn between 2D‐ and 3D‐derived EVs and a more selective sorting of these glycoproteins into EVs in 3D conditions. Further *O*‐glycoproteomic approaches should further validate these observations. Importantly, the predominant form of truncated *O*‐glycans identified in these EVs was the STn, independently of the cellular architecture.

Additionally, previous work from our group revealed that the most circulating glycoproteins presented STn instead of its non sialylated counterpart Tn.^[^ ^51^, ^52^ ^]^ It is described that non sialylated glycoproteins are more easily cleared from circulation by hepatic receptors.^[^ ^53^ ^]^ Moreover, STn is capable of binding to receptors present in immune cells, such as macrophages and dendritic cells, inducing immunosuppression.^[^ ^54^ ^]^ Therefore, the presence of the sialic acid on STn might have a protective role in cancer derived EVs in order to facilitate their circulation to distant sites while also inhibiting the immune system. Regarding other glycoforms, we observed alterations in branched and bisecting *N*‐glycans as well as *α*2,6‐sialylation when comparing 2D‐ and 3D‐derived EVs. Whereas 2D‐derived EVs reflected the glycan profile of the secreting cells, the profile of 3D‐derived EVs showed a more selective packaging. In terms of fucosylation, 3D‐derived EVs displayed an increase in fucosylation when compared to 2D‐derived EVs, mirroring their respective parental cells (Figure 3g). These observations are in agreement with our focusyltransferases gene expression analysis. The increased fucosylation by 3D has been observed in other studies using different cancer cell lines,^[^ ^4^, ^55^ ^]^ strongly suggesting that 3D architecture leads to an increase of fucosylated glycans. Further glycomic studies addressing the whole EV glycosylation profile are warranted, in order to validate its application in the clinical setting.

We also observed striking differences in lectin reactivity when comparing the WT and SC in 2D conditions, as we previously reported.^[^ ^19^ ^]^ On one hand, it is possible that truncation of *O*‐glycans may indirectly affect the synthesis of other glycosylation features, such as *N*‐glycans. This is to be expected, since the glycosylation machinery is an intricate and complex process, where shifting the balance toward the synthesis of a specific glycan may affect other structures.^[^ ^18^, ^56^ ^]^ On the other hand, in the 3D culture conditions it seems that these differences were decreased between WT and SC, both in spheroids and EVs. While spheroids demonstrate a high reactivity for L‐PHA, E‐PHA, and SNA, their respective EVs did not show the same high reactivity. This suggests that 3D cell culture conditions are not only able to impact the glycosylation machinery, but also EV glycoprotein packaging. These results suggest that cellular architecture might influence EV biogenesis and cargo sorting, which in turn could affect EV biology, uptake and biodistribution.

Glycans are one of the first molecules mediating the interaction between EV and recipient cells, and therefore the differences found in the glycosylation profiles between 2D‐ and 3D‐derived EVs may suggest different functional roles. Indeed, there has been a growing body of evidence suggesting the role of glycosylation in EV biology, namely affecting their uptake by recipient cells and regulating organ tropism.^[^ ^57^, ^58^, ^59^ ^]^ Considering the multitude of glycosyltransferases that regulate the glycosylation pathway,^[^ ^18^ ^]^ it is crucial to understand how cellular architecture affects the expression of these enzymes that would impact not only the cell glycome but also the glycome of their secreted EVs.

Considering glycans are known to modulate EV cargo sorting,^[^ ^60^, ^61^, ^62^ ^]^ alterations in the glycosylation profile might induce changes in the proteomic content of secreted EVs. Our proteomic analysis revealed that even though EVs derived from 2D and 3D SC cells shared most of the identified proteins, some are selectively enriched in EVs depending on the originating cell architecture. The enrichment of specific proteins observed in 2D‐ and 3D‐derived EVs might explain the distinct glycan profile present in these vesicles. The largest protein cluster found in 3D‐derived SC EVs includes many proteins, such as coagulation factors, SERPINs, or ITIH proteins, suggesting that these EVs might have potential to modulate the coagulation cascade. Induction of coagulation cascades was already observed in the cancer context^[^ ^63^ ^]^ and is associated with higher potential for metastasis formation and thromboembolism in patients.^[^ ^64^ ^]^ Notably, another important protein enriched in 3D‐derived SC EVs is the tissue factor (TF), a crucial initiator of the coagulation process.^[^ ^63^ ^]^ Interestingly, patients that have higher expression of these proteins demonstrated a decrease survival rate when compared to other clusters of patients. There is the possibility of increased chance of venous thromboembolism events in these patients, considering the physiopathology role of these proteins.^[^ ^64^, ^65^ ^]^ The fact that we found these proteins enriched 3D‐derived SC EVs, highlight the potential of this model in studying the EV role in the modulation of the coagulation process in cancer. Additionally, we observed an enrichment of proteins related to the regulation of complement cascade, namely C3, C4A, C5, and C7 in 3D‐derived EVs. It has already been reported that the presence of complement proteins in secreted EVs.^[^ ^66^ ^]^ It has been described that C3 and C5 (and their active forms, C3a and C5a), which are termed anaphylatoxins, are potent pro‐inflammatory molecules capable of reshaping the tumor microenvironment by enhancing tumor growth and metastatic potential.^[^ ^67^ ^]^ Although no association was found between these proteins and patient survival, these results suggest that 3D‐derived EVs may hold greater potential to modulate the complement cascade to sustain cancer progression. Furthermore, in EVs derived from both conditions, we found different ECM interacting proteins, highlighting the ECM modulatory role of EVs to induce premetastatic niches.^[^ ^13^ ^]^ Other groups have already reported alterations in the proteomic and transcriptomic cargo of EVs derived from 3D spheroids that better mimic in vivo shed EVs.^[^ ^2^, ^43^ ^]^ Altogether, the proteins identified in our EV samples allowed us to disclose subgroups of gastric cancer patients displaying different survival outcomes. It is plausible that EVs derived from 3D spheroids better mimic in vivo EV‐glycosylation, considering that the spheroids exhibit similar glycosylation profiles to patient tumors.^[^ ^4^ ^]^ Overall, it would be interesting in future studies to characterize the glycosylation features of the protein clusters identified to evaluate their biomarker potential in circulating EVs for patient prognosis and stratification in the clinical setting. Altogether, our proteomic analysis and EV‐glycosylation profiling showed that cellular architecture impacts the protein and glycan cargo of the secreted EVs, possibly leading to different biological effects. It is important to note that further functional studies are necessary to dissect the biological impact EVs isolated under different cell culture conditions in cancer.

## 5 Conclusion

In summary, we present data suggesting that different cellular organizations lead to distinct glycan and proteomic profiles of isolated EVs. The 3D cellular architecture promoted EV secretion compared to the conventional monolayer culture, and the EV‐glycan characterization unveiled different signatures, namely at the level of STn, fucosylation, *α*2,6‐sialylation, bisecting, and branched *N*‐glycan structures. These results highlight potential effects in EV biology, such as their uptake and biodistribution. In addition, quantitative proteomic analysis of EVs isolated from cells with different architectures revealed selective enrichment of specific proteins in each condition. Notably, differentially enriched proteins in EVs derived from spheroids established a denser network of protein interactions related to complement and coagulation cascade, ECM‐receptor interaction, focal adhesion, and cholesterol metabolism. Considering the key roles of EVs in mediating cell‐cell communication, our results point toward the importance of 3D cellular organization for future studies when assessing the glycosylation profile and biological role of isolated EVs from cancer cells.

## Conflict of Interest

The authors declare no conflict of interest.

## Author Contributions

Á.M.M. and T.M.L. contributed equally to this work. C.A.R. and D.F. conceptualized the hypothesis and designed the experimental approach. A.M.M., T.M.L., D.F., and J.P. performed the experiments. F.D. contributed to the establishment of the 3D cell culture methodology. F.P. contributed to the TCGA data analysis. H.O. performed mass spectrometry and proteomics. A.M.M., T.M.L., C.A.R., and D.F. wrote the manuscript. A.M.M. and T.M.L. prepared final images. All authors read, reviewed, and approved the final version of the manuscript.

## Supporting information

Supporting Information

## Data Availability

The data that support the findings of this study are openly available in ProteomeXchange at 10.6019/PXD035650, reference number PXD035650.
