# Supplementary material for: Differential Protein and Glycan Packaging into Extracellular Vesicles in Response to 3D Gastric Cancer Cellular Organization
Source: Adv Sci (Weinh). 2023 Jun 20;10(24):2300588. doi: 10.1002/advs.202300588 (PMC10460857; doi:10.1002/advs.202300588)
Supplement: Supplementary file 1 — Supporting Information [file ADVS-10-2300588-s001.pdf]

ADVANCED SCIENCE
Open Access

Supporting Information

for *Adv. Sci.*, DOI 10.1002/advs.202300588

Differential Protein and Glycan Packaging into Extracellular Vesicles in Response to 3D Gastric Cancer Cellular Organization

Álvaro M. Martins, Tânia M. Lopes, Francisca Diniz, José Pires, Hugo Osório, Filipe Pinto, Daniela Freitas and Celso A. Reis**

Supplementary material

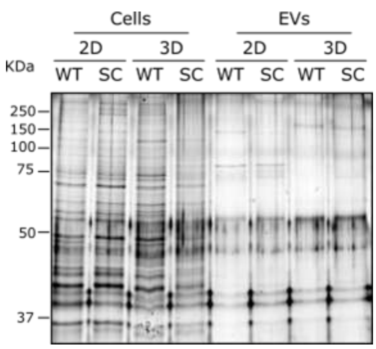

Supplementary material figure 1: Silver staining of 2D and 3D-cultured WT and SC cells and derived EVs

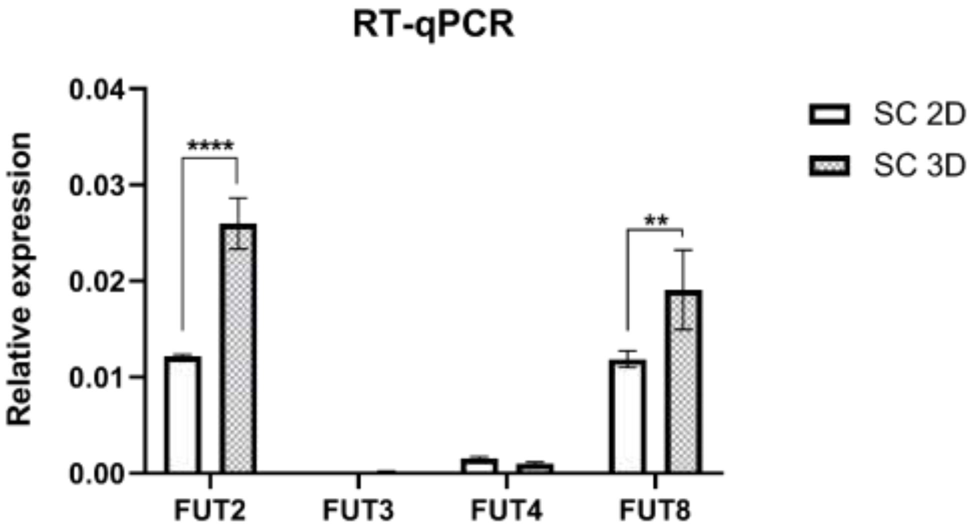

Supplementary material figure 2: Relative expression levels of *FUT2*, *FUT3*, *FUT4* and *FUT8* in both 2D and 3D SC cell models, using qRT-PCR assays. Results are shown as relative expression levels. Two independent experiments with at least two technical replicates per condition were performed. Results are shown as average ± SEM and two-way ANOVA was used for statistical analysis.

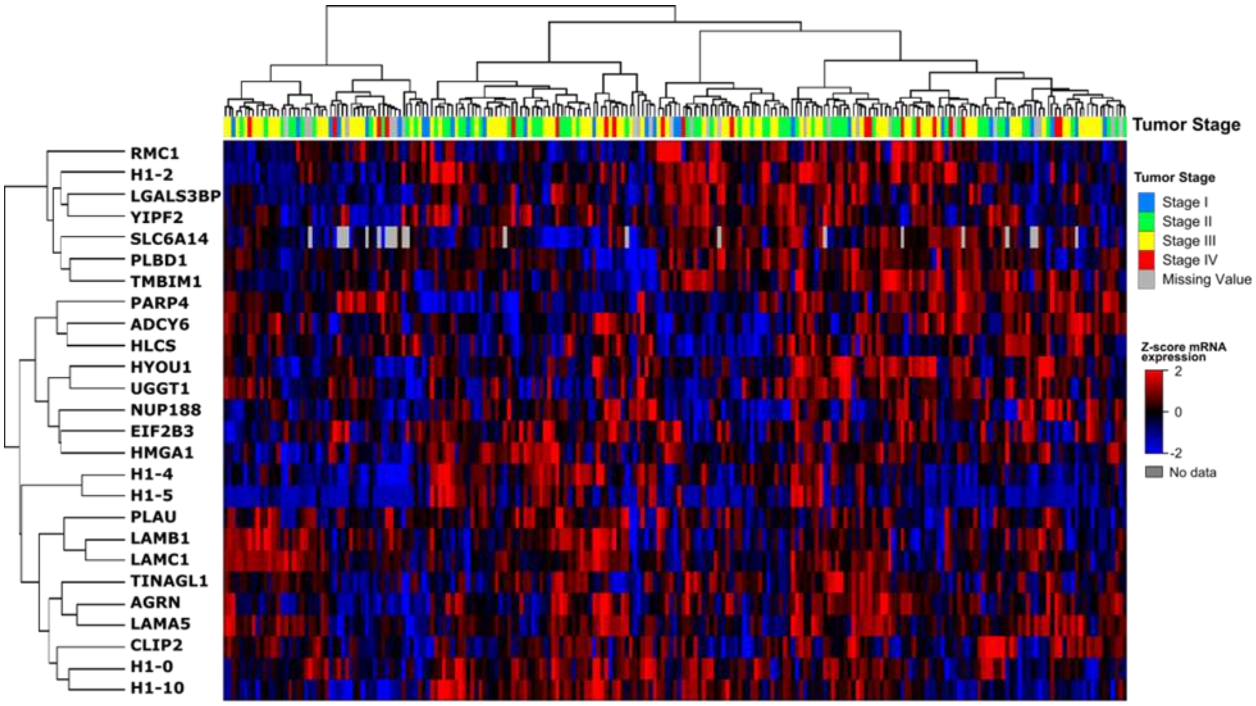

Supplementary material figure 3: Analysis of the transcriptomic profile from gastric cancer patients according to the proteins enriched in 2D-derived EVs. a) Heatmap of the transcriptomic profile of gastric cancer patients according to the proteins enriched in 2D-derived EVs. Only gastric cancer patients classified by the TCGA as chromosomal instability (CIN) molecular type were used, due to the CIN molecular characteristics found in MKN45 cell line. The z-score for mRNA expression and the covariate scale are represented on the right of the heatmap (missing values=grey)

Supplementary material table 1: Primer sequences for quantitative RT-PCR assays.

Gene	Species	Forward	Reverse
FUT2	Human	5'-GCGGCTAGCGAAGATTCAAG-3'	5'-TGATGTTGAGGCTAGCACTGGTA-3'
FUT3	Human	5'-CAAAATGCCAAGGGTGGACA-3'	5'-TTGG CCTCAATCAATCCTCCT-3'
FUT4	Human	5'-AAGCCGTTGAGGCGGTTT-3'	5'-ACAGTTGTGTATGAGATTTGG-3'
FUT8	Human	5'-CCATTTCAGGTTTGTTTGGTAG-3'	5'-ATTGGTCCCGCTTCTCACTT-3'
B-Actin	Human	5'-TACAGCTTCACCACCACAGC-3'	5'-AGTTTCATGGATGCCACAGG-3'
